# Zonally asymmetric changes in the Antarctic Circumpolar Current strength over the past million years

**DOI:** 10.1038/s41561-025-01901-2

**Published:** 2026-01-29

**Authors:** Shuzhuang Wu, Alain Mazaud, Elisabeth Michel, Michael P. Erb, Thomas F. Stocker, Helen Eri Amsler, Perig Le Tallec-Carado, Frank Lamy, Samuel L. Jaccard

**Affiliations:** 1 Institute of Earth Sciences, University of Lausanne, Lausanne, Switzerland; 2 State Key Laboratory of Deep-Sea Science and Intelligence Technology, Institute of Deep-Sea Science and Engineering, Chinese Academy of Sciences, Sanya, China; 3 Laboratoire des Sciences du Climat et de l’Environnement, CEA-CNRS-UVSQ, Université Paris Saclay, Gif-sur-Yvette, France; 4 School of Earth Science and Environmental Sustainability, Northern Arizona University, Flagstaff, AZ USA; 5 Oeschger Centre for Climate Change Research, University of Bern, Bern, Switzerland; 6 Institute of Geological Sciences, University of Bern, Bern, Switzerland; 7 University of Bordeaux, Bordeaux, France; 8 Alfred Wegener Institute, Helmholtz-Centre for Polar and Marine Research, Bremerhaven, Germany; 9 MARUM–Center for Marine Environmental Sciences, Bremen, Germany; 10 Present Address: University Library Bern, University of Bern, Bern, Switzerland

**Keywords:** Palaeoceanography, Palaeoclimate

## Abstract

The Antarctic Circumpolar Current (ACC) plays a central role in regulating the global ocean circulation, climate and Antarctic Ice Sheet dynamics. Yet the spatiotemporal variability of the ACC during the Pleistocene remains poorly constrained. Here we reconstruct ACC flow-speed variation using a meridional transect of sediment cores from the Indian sector of the Southern Ocean. Our results reveal zonally asymmetric changes in ACC strength across the Southern Ocean on orbital timescales over the past one million years; the ACC intensified in the South Indian Ocean but weakened in the South Pacific during glacial and low-obliquity periods, with the opposite pattern during interglacial and high-obliquity periods. These anti-phased changes probably reflect an integrated response to bathymetric constraints, shifts in the Southern Hemisphere westerlies, sea-ice extent, buoyancy forcing and current confluence. Such zonally asymmetric and anti-phased ACC dynamics persisted during warmer-than-present intervals of the Pleistocene, offering a potential analogue for future anthropogenic warming—albeit under fundamentally different boundary conditions.

## Abstract (web summary)

Anti-phased changes in Antarctic Circumpolar Current strength between the Indian and Pacific sectors of the Southern Ocean occurred on orbital timescales over the past one million years, linked to glacial cycles and obliquity forcing, according to proxy records and modelling.

## Main

The Antarctic Circumpolar Current (ACC) is the largest current system in the world oceans, yet it remains among the most poorly understood components of the global ocean circulation. The ACC plays an essential role in the climate system as it regulates interbasin exchange of physical, chemical and biological properties, thus enabling a truly global overturning circulation^1,2^. The meridional transport of water masses across the ACC leads to poleward heat advection and intense upwelling of CO_2_^−^ and nutrient-rich subsurface waters along tilted surfaces of constant density (isopycnals). By modulating the poleward transport of heat and the advection of warm Circumpolar Deep Water (CDW), the ACC influences the extent and stability of the Antarctic cryosphere^3–6^.

**Fig. 1:**
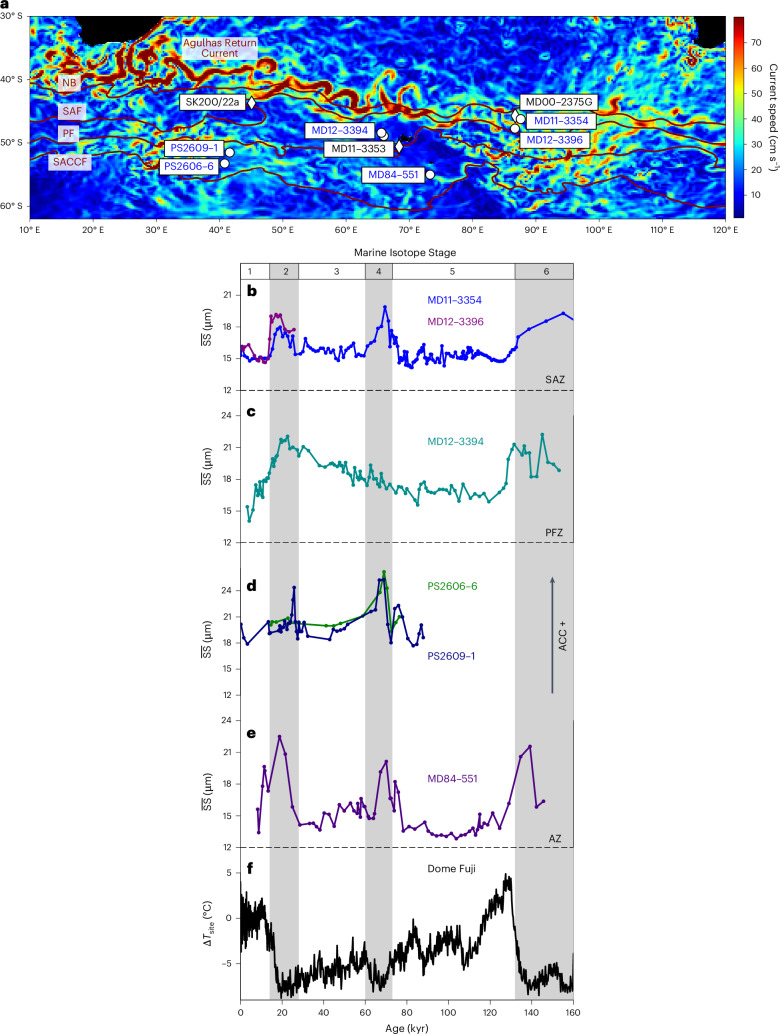
Changes in ACC flow speed along a north–south transect from the SAZ to the AZ in the South Indian Ocean over the last glacial cycle. **a**, Modern surface ACC velocity map. The oceanic fronts are as derived from in situ measurements and satellite altimetry^10^. Core locations are marked by white circles (this study, based on $\overline{{\rm{SS}}}$ proxy) and diamonds (based on magnetic grain size; Extended Data Fig. 3). **b**, ACC strength variations in the SAZ between the STF and SAF (MD11-3354 and MD12-3396, eastern Kerguelen Plateau). **c**, ACC strength variations in the Polar Frontal Zone (PFZ) between the SAF and PF (MD12-3394, western Kerguelen Plateau). **d**,**e**, ACC strength variations in the AZ between the PF and SACCF (PS2606-6 and PS2609-1, Conrad Rise; MD84-551, southern Kerguelen Plateau). **f**, Antarctic temperature record from the Dome Fuji ice core^47^. Vertical grey bars mark the glacial periods during Marine Isotope Stages 2, 4 and 6. Basemap in **a** from GMRT^61^ with data from GODAS^62^ under a Creative Commons license CC BY 4.0.

**Extended Data Fig. 1:**
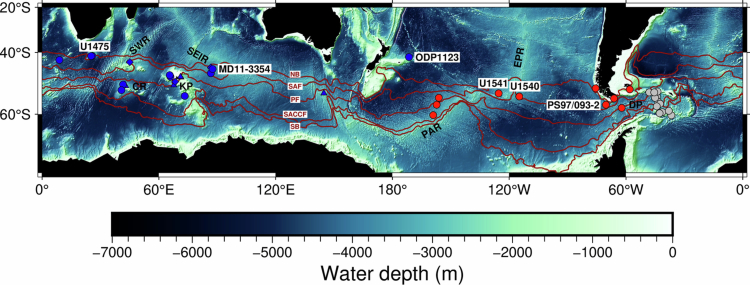
Bathymetric map of the South Indian Ocean. The oceanic fronts (red lines) are indicated after Park et al^10^. NB, Northern Boundary; SAF, Subantarctic Front; PF, Polar Front; SACCF, Southern ACC Front; SB, Southern Boundary. The symbols used are dots for SS-based, diamonds for magnetic grain-based, and triangles for benthic foraminifer-based reconstructions^78,80–83^ (see Supplementary Table 1). SWR, Southwest Indian Ridge; SEIR, Southeast Indian Ridge; CR, Conrad Rise; KP, Kerguelen Plateau;EPR, East Pacific Rise; PAR,Pacific Antarctic Ridge; DP, Drake Passage. Bathymetry map from GMRT^62^ under a Creative Commons license CC BY 4.0.

The ACC is a deep-reaching eastward flow steered by bathymetry and continental topography. Its trajectory extends southeastwards from the Indian Ocean into the southeast Pacific before undergoing a pronounced northward deflection upon exiting the Drake Passage into the western Atlantic basin (Fig. 1a and Extended Data Fig. 1). More than 95% of its transport is confined within the three major circumpolar fronts^7,8^: the Subantarctic Front (SAF), the Polar Front (PF), and the Southern ACC Front (SACCF) from north to south^9,10^. Modern ACC dynamics are governed primarily by wind stress, buoyancy forcing and mesoscale eddies, with far-reaching impacts on the carbon cycle and global climate system, both today and in the geological past^11–13^.

Proxy-based reconstructions of ACC variability during the Pleistocene Epoch remain insufficiently constrained (both temporally and geographically), limiting our ability to resolve the main drivers of change. Previous investigations have typically assumed zonally integrated changes in ACC strength through time^14–17^. In the South Pacific, sedimentary records spanning the SAF and PF signal a coherent glacial–interglacial pattern, with weaker ACC strength during glacials over the past ~350 thousand years (kyr; ref. ^18^). By contrast, only minor variations or front-related shifts in ACC strength are recorded in the Scotia Sea (southwestern Atlantic)^15,19^. On the contrary, evidence for stronger glacial ACC flow was documented in the southeastern Atlantic^20^ and South Indian Ocean^14,21^. These findings suggest that orbital-scale changes in ACC strength were not zonally homogeneous.

Because both local and regional effects must be considered to reconstruct past ACC dynamics, large-scale insights cannot be reliably inferred from a single core location. The scarcity of well-constrained sediment records, especially in the South Indian Ocean, further limits robust assessment of ACC variability across the entire Southern Ocean. To address this gap, we use a transect of six marine sediment cores spanning all major oceanic fronts in the South Indian Ocean to reconstruct the spatiotemporal variability in ACC strength on multiple timescales (Fig. 1 and Extended Data Fig. 1). We use the mean grain size of non-cohesive siliciclastic sortable silt ($\overline{{\rm{SS}}}$, 10–63 μm; Extended Data Fig. 6) as a proxy of near-bottom current speed^22^. Modern observations suggest that the ACC influences the full water column, with near-bottom current velocities reflecting its deep-reaching structure^23^. Mesoscale eddy variability drives interannual to decadal ACC variability, with eddy kinetic energy decaying exponentially with depth and potentially offsetting wind forcing under eddy saturation^24,25^. On millennial to orbital timescales, however, the $\overline{{\rm{SS}}}$ proxy reflects a scalar, water-column-integrated current speed, capturing the combined effects of wind, baroclinic and eddy-driven forcing^15,18^.

## Coherent changes in ACC strength across fronts

Our meridional transect of sediment records spanning the past 160 kyr reveals low and stable $\overline{{\rm{SS}}}$ values during interglacials, in contrast to higher and more variable values during glacials (Fig. 1). These results suggest a spatially coherent increase in ACC flow speed across a broad latitudinal range under cold climate conditions. Evidence for stronger glacial ACC is further supported by high $\overline{{\rm{SS}}}$ values on the Agulhas Plateau^21^ as well as coarser magnetic grain sizes in the South Indian Ocean^14,26^ (Extended Data Fig. 3) and enhanced sediment focusing along the eastern Kerguelen Plateau^27^. Together, these lines of evidence point to a strengthening of the ACC flow during glacial periods. Conversely, our records document a slow-down of the ACC across most oceanic fronts during the last interglacial and Holocene. Relative to the Holocene mean, glacial ACC strength was enhanced by ~13–38% in the Subantarctic Zone (SAZ), ~26–43% in the Polar Frontal Zone and ~10–44% in the Antarctic Zone (AZ) (Fig. 1).

Reconstructions of sea surface temperature indicate that oceanic fronts may have shifted relative to the bathymetry^28,29^, which may have influenced ACC dynamics on orbital timescales. A recent composite record from the northern boundary (NB) of the Agulhas Plateau indicates that frontal shifts may have modulated glacial–interglacial variations in ACC strength^21^. By contrast, our transect records reveal coherent amplitude changes in ACC strength across a broad latitudinal and longitudinal range in the Indian sector of the Southern Ocean, including sites to the west of Conrad Rise as well as to the west and east of the Kerguelen Plateau (Fig. 1 and Extended Data Fig. 1). These results indicate that oceanic frontal movements, steered by the local bathymetry, may have exerted only a secondary control on ACC variability in the pelagic South Indian Ocean on glacial–interglacial timescales. These findings are consistent with results from a similar transect in the central South Pacific^18^.

**Fig. 2:**
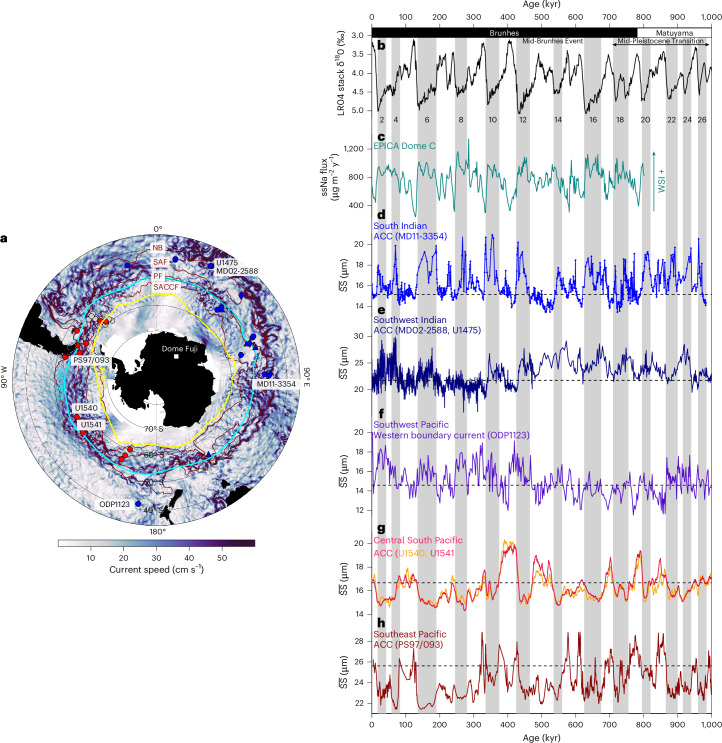
Zonally asymmetric changes in ACC strength across the Southern Ocean. **a**, Proxy-based reconstructions of the ACC variability across the Southern Ocean based on sediment cores retrieved nearby or southwards of the NB. Blue markers indicate a weakened ACC strength during interglacial periods; red dots denote a strong ACC strength. Grey dots indicate no substantial changes in ACC flow speed. The symbols used are dots for SS-based, diamonds for magnetic grain-based, and triangles for benthic foraminifer-based reconstructions (Supplementary Table 1). Yellow and blue lines indicate winter sea ice (WSI) edge with 15% sea-ice concentration at modern (M-WSI) and the Last Glacial Maximum (LGM-WSI), respectively^36,37^. **b**, Benthic foraminifera oxygen isotope LR04 stack^63^. **c**, Sea-salt sodium (ssNa) flux from the EPICA Dome C core, a proxy for WSI extent^64^. **d**, ACC strength variations at site MD11-3354 (blue) in the South Indian. **e**, ACC strength variations in the Southwest Indian, the Agulhas Plateau Composite, including sediment cores MD02-2588 and IODP U1475 (dark blue)^21^. **f**, Western boundary current variations at site ODP1123 (purple) east of New Zealand in the southwest Pacific^55^. **g**, ACC strength variations at IODP sites U1540 (orange), U1541 (red) in the Central South Pacific^18^. **h**, ACC strength variations at site PS97/093 (maroon) in the southeast Pacific^30^. The dashed lines indicate the Holocene mean level of the ACC strength. Vertical grey bars and even numbers mark the glacial stages. Basemap in **a** from GMRT^62^ with data from GODAS^61^ under a Creative Commons license CC BY 4.0.

**Extended Data Fig. 2:**
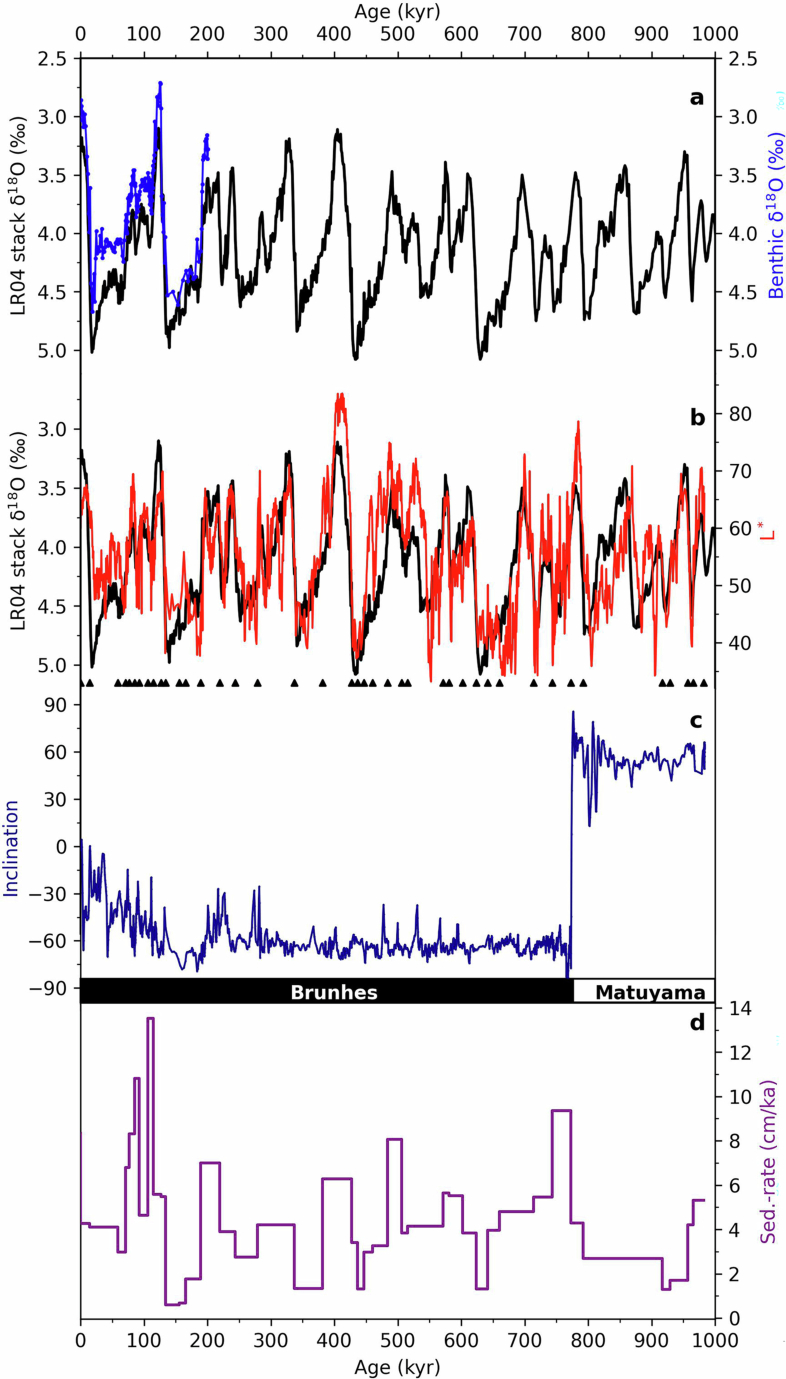
Stratigraphy for MD11-3354. **a**, Benthic δ^18^O record from MD11-3354 (blue) tuned to the LR04 δ^18^O stack^63^ (black). **b**, Reflectance L* record (red) tuned to the LR04 δ^18^O stack^63^ (black). Triangles mark the tuning points. **c**, Inclination record from MD11-3354. **d**, Sedimentation-rate record from MD11-3354.

**Extended Data Fig. 3:**
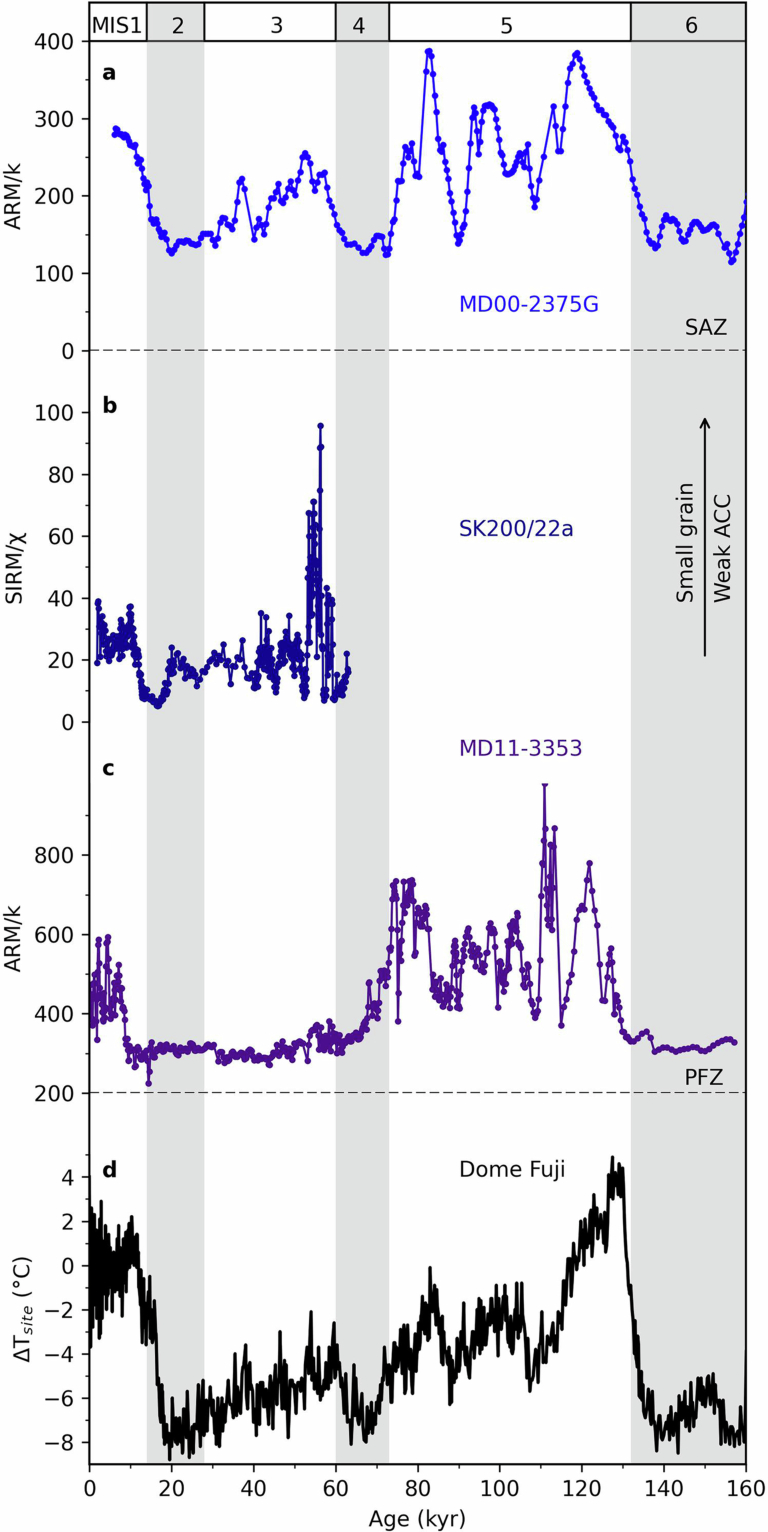
Reconstructions of ACC strength variations based on magnetic grain size from the SAZ to PFZ in the Indian sector over the last glacial cycle. **a**, ACC strength variations in the SAZ (MD00-2375G, eastern Kerguelen Plateau)^14^. **b**, ACC strength variations in the PFZ (SK200/22a, Crozet Plateau)^78^. **c**, ACC strength variations in the PFZ (MD11-3353, Kerguelen Plateau)^26^. **d**, Antarctic temperature record from the Dome Fuji ice core^47^. Increases in ARM/κ and SIRM/χ indicate smaller grain size and weaker ACC strength^14,26,78^.

Beyond the last glacial cycle, our high-resolution $\overline{{\rm{SS}}}$ record reveals coherent glacial–interglacial oscillations in ACC strength in the southeastern Indian Ocean over the past one million years (Myr), with consistently stronger glacial ACC flow relative to interglacials (Fig. 2d and Extended Data Fig. 2). On longer timescales, the magnitude of these fluctuations increased across the Mid-Brunhes Event (MBE; ~430 kyr), after which glacial–interglacial fluctuations became more pronounced (Fig. 2d). Large amplitude changes (~20–47%) occurred during the most recent four glacial cycles, contrasting with modest variations (~12–30%) in ACC strength between Marine Isotope Stages 14 and 22. During post-MBE glacials, ACC strength in the Indian sector of the Southern Ocean reached up to 140% of its Holocene mean, while interglacials were marked by ACC flow speeds similar to the Holocene mean (Fig. 2d). Taken together, our records indicate persistently stronger glacial ACC strength over the past 1 Myr across all frontal zones in the Indian sector of the Southern Ocean.

## Anti-phased changes in ACC strength across the Southern Ocean

To further assess spatiotemporal variability of the ACC, we compiled reconstructions spanning all sectors of the Southern Ocean. Our compilation shows zonally asymmetric changes in ACC strength over the past 1 Myr: it weakened in the Indian sector while it strengthened in the Pacific sector during interglacial periods, and vice versa during glacials^18,21,30^ (Fig. 2). Modern observations also exhibit zonal asymmetry in ACC transport on interannual timescales, but without sustained anti-phasing across the Southern Ocean^31^. This contrast indicates that the persistent anti-phased variability observed in the Pleistocene reflects an integrated response to long-term climate forcings rather than a transient expression of short-term processes.

**Extended Data Fig. 4:**
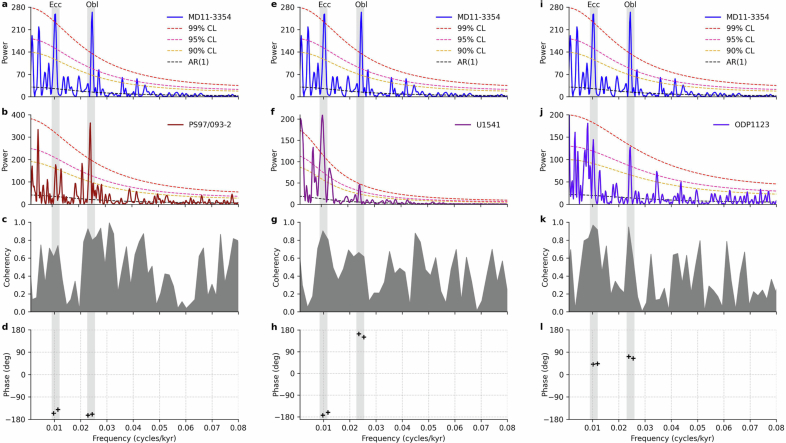
Comparisons of spectral and phase pattern. **a, b** Spectral analysis of $\overline{{\rm{SS}}}$ records with the corresponding phase and coherency spectrum from sites MD11-3354 (blue) in the South Indian Ocean and PS97/093 (red) in the southeast Pacific^30^. It shows high coherency at eccentricity and obliquity bands **c**, with anti-phase variations between of MD11-3354 and PS97/093 (**d**). **e, f** Spectral analysis of $\overline{{\rm{SS}}}$ records with the corresponding phase and coherency spectrum from sites MD11-3354 (blue) in the South Indian Ocean and IODP U1541 (purple) in the central South Pacific^18^. It shows high coherency at eccentricity and obliquity bands (**g**) with anti-phase variations between of MD11-3354 and IODP U1541 (**h**). **(i, j**) Spectral analysis of $\overline{{\rm{SS}}}$ records with the corresponding phase and coherency spectrum from sites MD11-3354 (blue) and ODP1123 at east of New Zealand^55^ (blue purple). It shows high coherency at eccentricity and obliquity bands (**k**) with in-phase variations between of MD11-3354 and ODP1123 (**l**). In all spectral power plots, the confident levels are 99% (red), 95% (pink) and 90% (orange). Grey bars indicate the eccentricity (Ecc) and obliquity (Obl) cycles.

Spectral analyses reveal significant variance at eccentricity (~100 kyr) and obliquity (~41 kyr) bands in both the Indian and Pacific sectors of the Southern Ocean (99% confidence level; Extended Data Fig. 4a,b,f). By contrast, precession-related cycles are not statistically significant (<95% confidence level; Extended Data Fig. 4a,b,f). These results suggest that past changes in ACC strength were modulated primarily by glacial–interglacial climate dynamics and obliquity forcing.

On glacial–interglacial timescales, asymmetric ACC variability probably reflects the combined influence of the Southern Hemisphere Westerly Winds (SWW), sea-ice extent and meridional density gradients. Although the magnitudes of past SWW shifts remain uncertain, reconstructions and modelling simulations are consistent with an equatorward displacement during glacial times^11,32–34^. The ocean fronts shift was spatially heterogeneous, with only a minor shift (2–5°) in the South Indian Ocean but a more substantial migration (5–10°) in the Southeast Pacific^28,29,34,35^. At the same time, winter sea ice in the South Pacific may have extended to ~51–55° S during the last ice age^35–37^ (Fig. 2), overlapping with the modern mean ACC latitude (~58° S) and thereby dampening wind stress on the ocean surface, weakening Pacific ACC strength. By contrast, sea-ice expansion in the South Indian Ocean was confined to ~50° S during glacial maxima^35,36^ (Fig. 2), leaving the more northerly ACC (~45° S) directly exposed to equatorward-shifted SWW and enhanced meridional density gradients. These conditions probably steepened isopycnal slopes and thereby intensified glacial ACC flow in the Indian sector of the Southern Ocean.

**Fig. 3:**
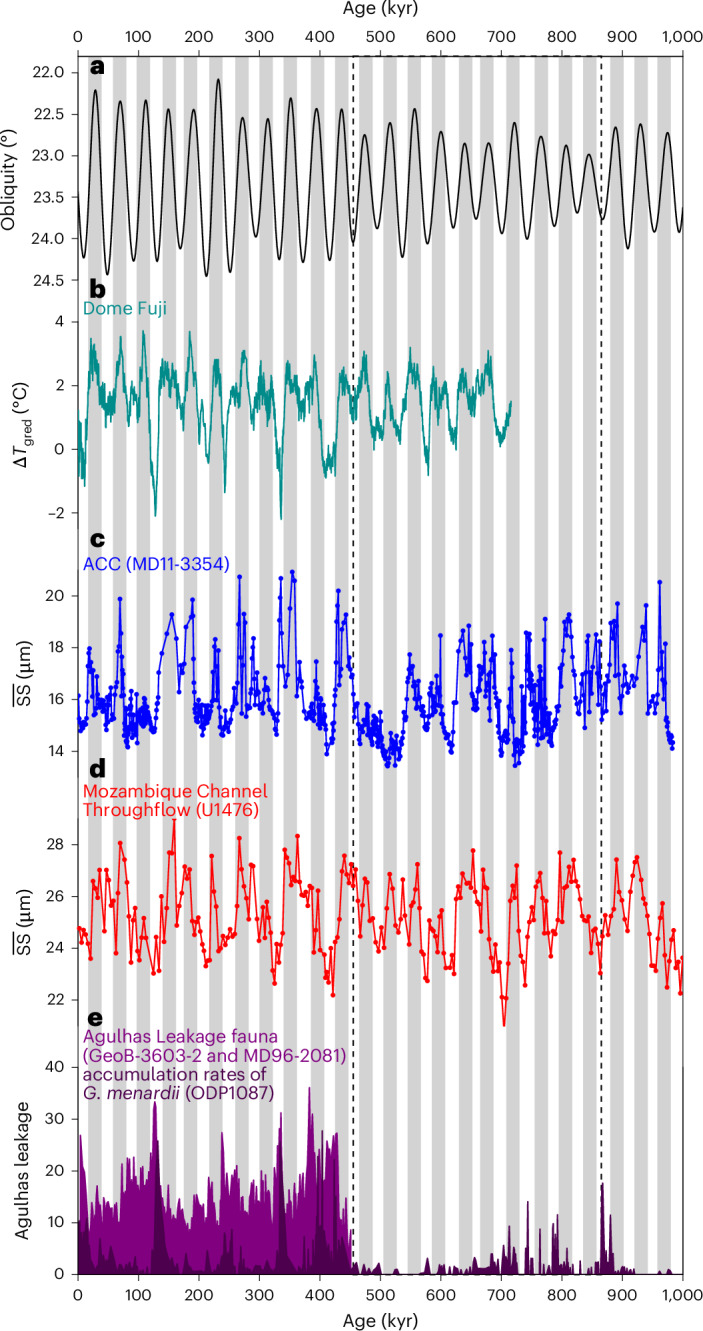
Mechanistic forcings on the ACC variations. **a**, Obliquity with inverted *y* axis. **b**, Temperature gradient between source and site from Dome Fuji ice core^47^. **c**, ACC strength variations at site MD11-3354 (blue) in the South Indian. **d**, Mozambique Channel Throughflow (red) variations at site IODP U1476^41^. **e**, Agulhas Leakage proxies using Agulhas Leakage fauna (light purple) counts from cores GeoB-3603 and MD96-2081^40^ and accumulation rates (number cm^−2^ kyr^−1^, dark purple) of *Globorotalia menardii* from ODP1087^39^. Dashed frame indicates low-amplitude variations of obliquity and ACC strength. Grey bars mark obliquity minima.

The equatorward shift of the SWW and associated oceanic fronts may have reduced the Agulhas leakage by limiting the transport of warm surface waters from the Indian Ocean into the South Atlantic^38–40^. Simultaneously, an intensified Mozambique Channel Throughflow could have reinforced the Agulhas Return Current^41^ (Fig. 3d,e), whose powerful confluence probably accelerated ACC strength in the South Indian Ocean (Fig. 3c). Reduced Indian–Atlantic water exchange may have led to the accumulation of warm, saline waters in the South Indian Ocean, where the dominant thermal expansion of warming outweighed the opposing effect of salinity on density, enhancing the meridional density gradients during glacial periods^11,42^. These warm surface waters were probably advected by the Agulhas Return Current, consistent with glacial surface–subsurface temperature difference exceeding 9 °C in the study region^43^. Eddy-resolving simulations further suggest that enhanced surface buoyancy forcing leads to an increase in ACC transport steered by a steeper meridional density gradient and a deeper thermocline^44–46^. Accordingly, increased meridional density gradients and intensified surface heat flux driven by sea-ice expansion and warm-water intrusion would have intensified buoyancy forcing and reinforced ACC flow in the South Indian Ocean during glacials (Fig. 2). By contrast, in the South Pacific, limited subtropical warm-water input probably produced cooler, denser waters in the northern ACC, reducing the meridional density gradients and weakening buoyancy forcing. This mechanism contributed to a weaker ACC in the South Pacific during glacial periods^17,18,30^.

**Extended Data Fig. 5:**
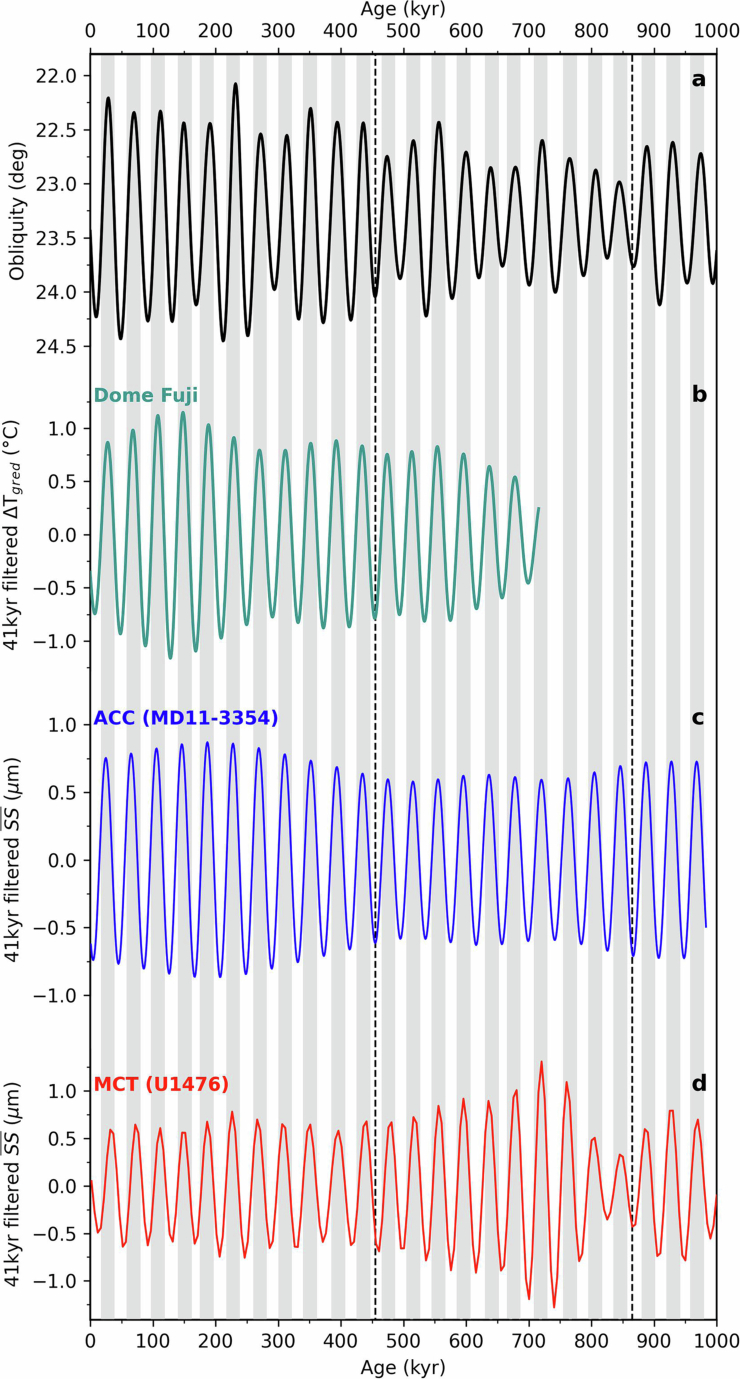
Obliquity components of climate records. **a**, Obliquity with inverted y-axis. **b**, The obliquity component of temperature gradient from Dome Fuji ice core^47^. **c**, The obliquity component of ACC strength in the Indian sector of the Southern Ocean. **d**, The obliquity component of MCT strength^41^. Dashed frame indicates low amplitude variations of obliquity and ACC strength.

**Extended Data Fig. 6:**
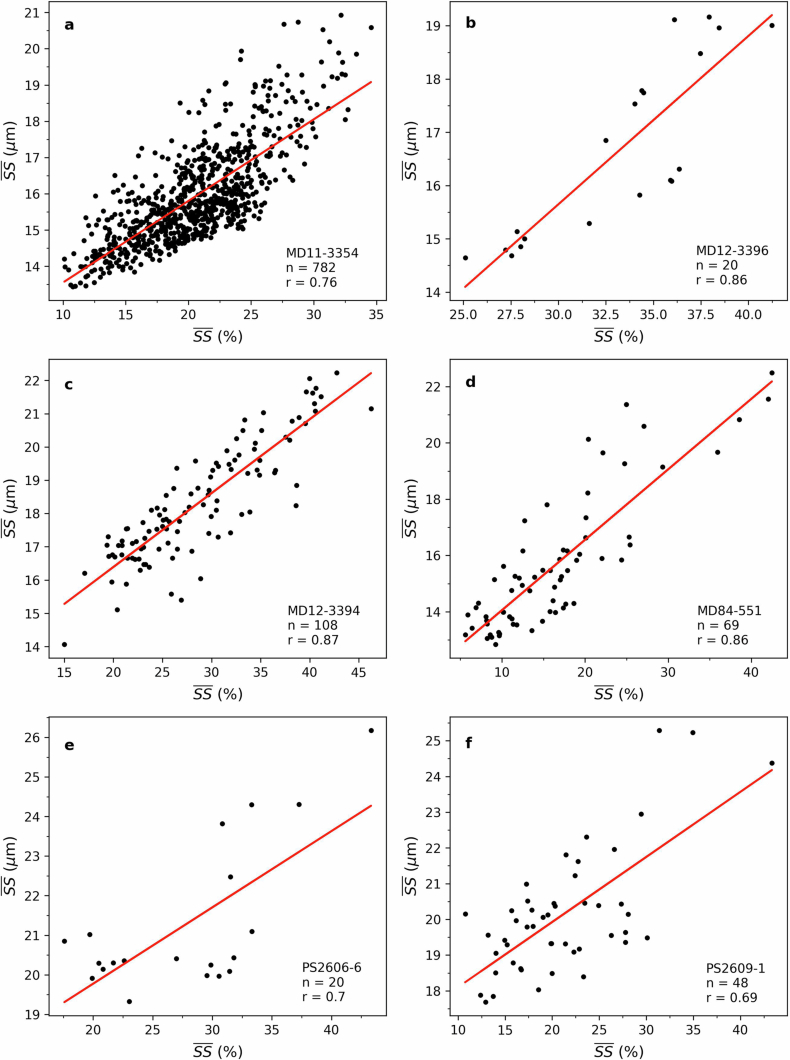
Comparisons of $\overline{{\rm{SS}}}$ and $\overline{{\rm{SS}}}$ percentage in this study. **a**, Site MD11-3354. **b**, Site MD12-3396. **c**, Site MD12-3394. **d**, Site MD84-551. **e**, Site PS2606-6. **f**, Site PS2609-1. All records show a positive correlation, indicating that the $\overline{{\rm{SS}}}$ is predominantly current-controlled^79^.

Superimposed on these glacial–interglacial variabilities, zonally asymmetric changes in ACC strength also occur on obliquity timescales (Extended Data Fig. 4a,b,f). Cross-spectral analyses reveal that an antiphase relationship between obliquity and ACC strength in the South Indian Ocean, with a stronger flow during low-obliquity intervals and a weaker flow during high-obliquity intervals (Fig. 3a,c and Extended Data Fig. 5). This antiphase pattern persisted through the Middle and Late Pleistocene, encompassing the MBE and later part of the Mid-Pleistocene Transition (Fig. 3c). Obliquity-paced (41 kyr) fluctuations in ACC strength in the South Indian intensified after the MBE, indicating higher sensitivity to obliquity forcing, probably mediated by its dominant control on meridional temperature gradients and Southern Ocean sea-ice variability^47–49^ (Fig. 3a,c and Extended Data Fig. 5). By contrast, changes in ACC strength in the South Pacific are in phase with obliquity, with a stronger ACC flow during high-obliquity intervals and a weaker flow during low-obliquity intervals^18,30^ (Extended Data Fig. 4). These asymmetric Indo–Pacific responses at obliquity scale indicate distinct mechanistic forcings and regionally differentiated impacts on ACC variability.

To explore the mechanisms underlying the obliquity-paced ACC variability, we analysed simulations performed with the National Center for Atmospheric Research (NCAR) Community Earth System Model version 1.2 (CESM1.2)^50^. In these experiments, orbital forcing was driven solely by changes in obliquity, with conditions set to either minimum or maximum obliquity while maintaining other boundary conditions unchanged.

**Fig. 4:**
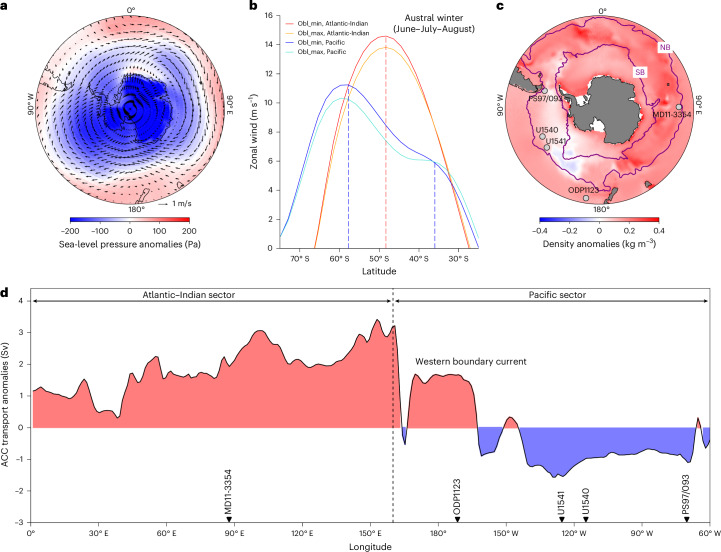
Simulated climate changes resulting from minimum and maximum obliquity in NCAR CESM1.2. Anomalies are presented by obliquity minimum minus obliquity maximum. **a**, Sea-level pressure anomalies (colour) and zonal wind anomalies (black vectors), showing positive Southern Annual Mode-like responses with low sea-level pressure at the South Pole and higher sea-level pressure in the mid-latitudes. **b**, Zonal wind strength at 850 hPa during austral winter in the Atlantic–Indian sector (red and orange) and Pacific sector (blue and cyan) during minimum and maximum obliquity (obl_min and obl_max), respectively. Vertical dashed lines mark the peaks of the jet stream. **c**, Integrated density anomalies. Basemaps in **a** and **c** based on simulation datasets from NCAR CESM^50^. SB, Southern boundary. These boundaries are defined from satellite altimetry and in situ observations^10^. **d**, ACC transport (42° S–58° S) anomalies across the Atlantic–Indian sector (0°–160° E) and Pacific sector (160° E–60° W) of the Southern Ocean. Panels **a** and **c** adapted from ref. ^49^ under a Creative Commons license CC BY 4.0.

**Extended Data Fig. 7:**
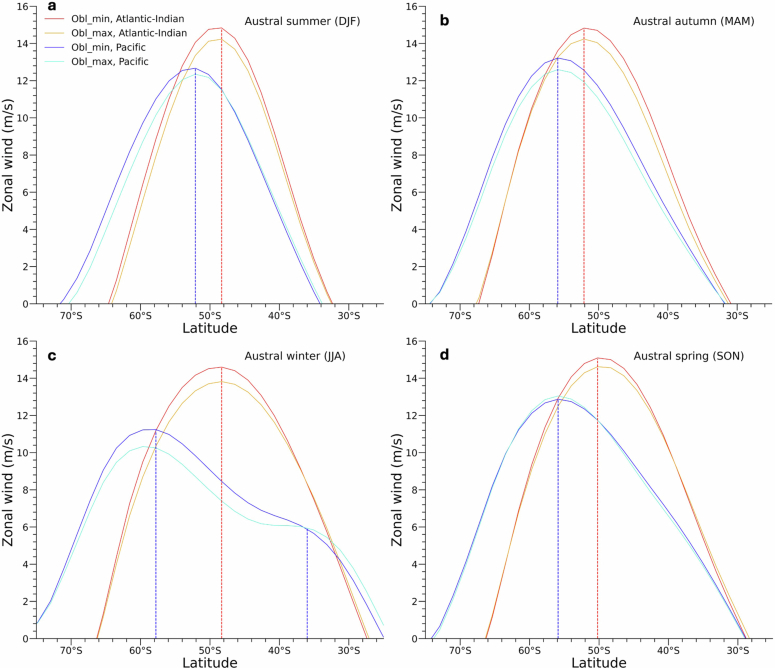
Simulated seasonal changes in zonal wind strength across the Southern Ocean during minimum obliquity relative to maximum obliquity. **a**, Austral summer (December to February). **b**, Austral autumn (March to May). **c**, Austral winter (June to August). **d**, Austral spring (September to November).

**Extended Data Fig. 8:**
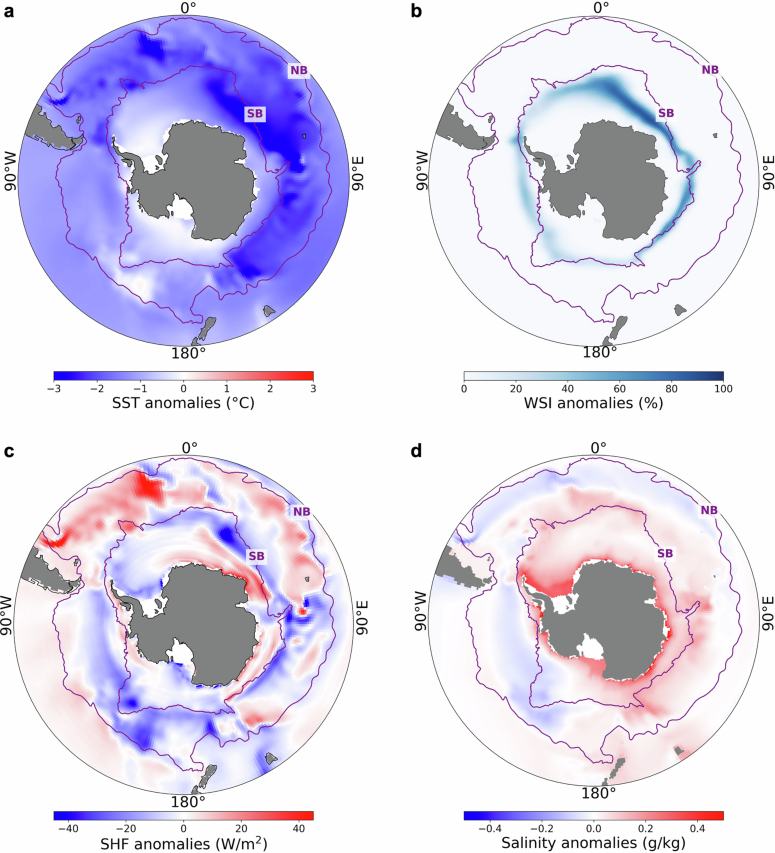
Simulated results by obliquity minimum relative to obliquity maximum. **a**, Sea surface temperature (SST) anomalies. **b**, Winter sea ice anomalies. **c**, Surface heat flux (SHF) anomalies. **d**, Integrated salinity anomalies. NB, Northern Boundary. SB, Southern Boundary. The oceanic boundaries (purple lines) are indicated after Park et al.^10^. Basemaps were based on simulation datasets from NCAR CESM^50^. Figure adapted from ref. ^49^ under a Creative Commons license CC BY 4.0.

**Extended Data Fig. 9:**
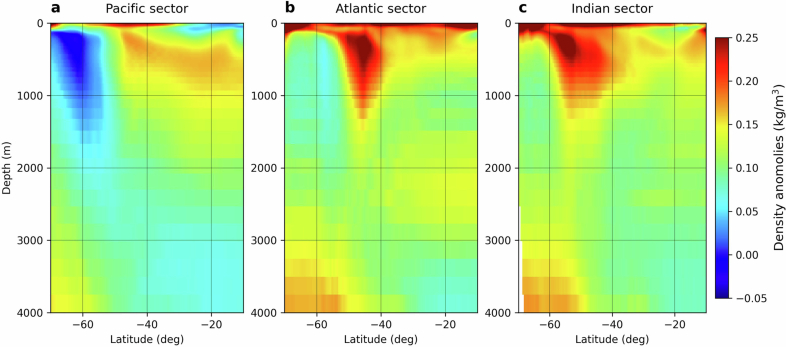
Southern Ocean density anomalies during obliquity minimum relative to maximum. **a**, Pacific sector; **b**, Atlantic sector; **c**, Indian sector. A decrease in the meridional density gradient within the South Pacific, contrasting with an increase across the Atlantic-Indian sectors of the Southern Ocean.

Our simulations reveal positive Southern Annular Mode-like responses, characterized by intensified SWW over the Southern Ocean (Fig. 4a). Stronger SWW during low-obliquity intervals are further supported by enhanced meridional temperature gradients as indicated by the larger temperature contrast between the moisture source region and the Dome Fuji ice core site under low obliquity compared with high obliquity^47^ (Fig. 3b). Specifically, a single jet stream intensified over the Atlantic–Indian sector for all seasons (Fig. 4b and Extended Data Fig. 7), accelerating the ACC in the South Indian Ocean during obliquity minima relative to maxima (Fig. 3c). Concurrently, sea-ice expansion in the South Indian Ocean was probably confined south of the ACC, thereby enhancing meridional density gradients and surface heat flux during low-obliquity intervals (Fig. 4c and Extended Data Figs. 8 and 9). These conditions steepened isopycnal slopes, deepened the thermocline and collectively strengthened buoyancy forcing^45^, which amplified ACC transport in this sector (Fig. 4d).

By contrast, the South Pacific jet streams exhibit pronounced seasonal variability with a stronger split jet structure during austral winter (Fig. 4b and Extended Data Fig. 7), consistent with earlier observations of zonally heterogeneous changes in the SWW over the past 1 Myr (ref. ^34^). During low-obliquity intervals, the strong split jet configuration probably misaligned peak SWW with the main ACC trajectory. Combined with expanded sea ice, this would have reduced the efficacy of SWW forcing on the ocean surface, thus weakening the ACC in the South Pacific^11,18^. Furthermore, reduced meridional density gradients and diminished surface heat flux during low-obliquity intervals (Fig. 4c and Extended Data Figs. 8 and 9) would have decreased buoyancy forcing^45^ and further weakened ACC strength in the South Pacific (Fig. 4d).

## Implications of Pleistocene ACC dynamics

Zonally asymmetric changes in ACC strength were probably mechanistically coupled to the variability of the East and West Antarctic Ice Sheets (EAIS and WAIS). During the Pleistocene interglacials, a weakened ACC in the Atlantic and Indian sectors of the Southern Ocean would have reduced southward heat transport and advection of warm CDW onto the EAIS continental shelf, contributing to its stability^51^. Conversely, a stronger ACC in the South Pacific may have enhanced poleward heat transport and CDW advection into the Ross Sea, potentially triggering the retreat or collapse of the WAIS^5,18,52^. This asymmetric pattern is also expressed at obliquity timescales, with intensified Pacific ACC increasing ocean heat delivery to the Ross Sea coinciding with WAIS retreat during high-obliquity intervals^5,18,30^. Taken together, these findings suggest that zonally asymmetric changes in ACC strength, in concert with other climatic forcings, have long modulated the EAIS and WAIS dynamics and will certainly continue to influence the ice-sheet variability under future climate changes^53,54^.

**Extended Data Fig. 10:**
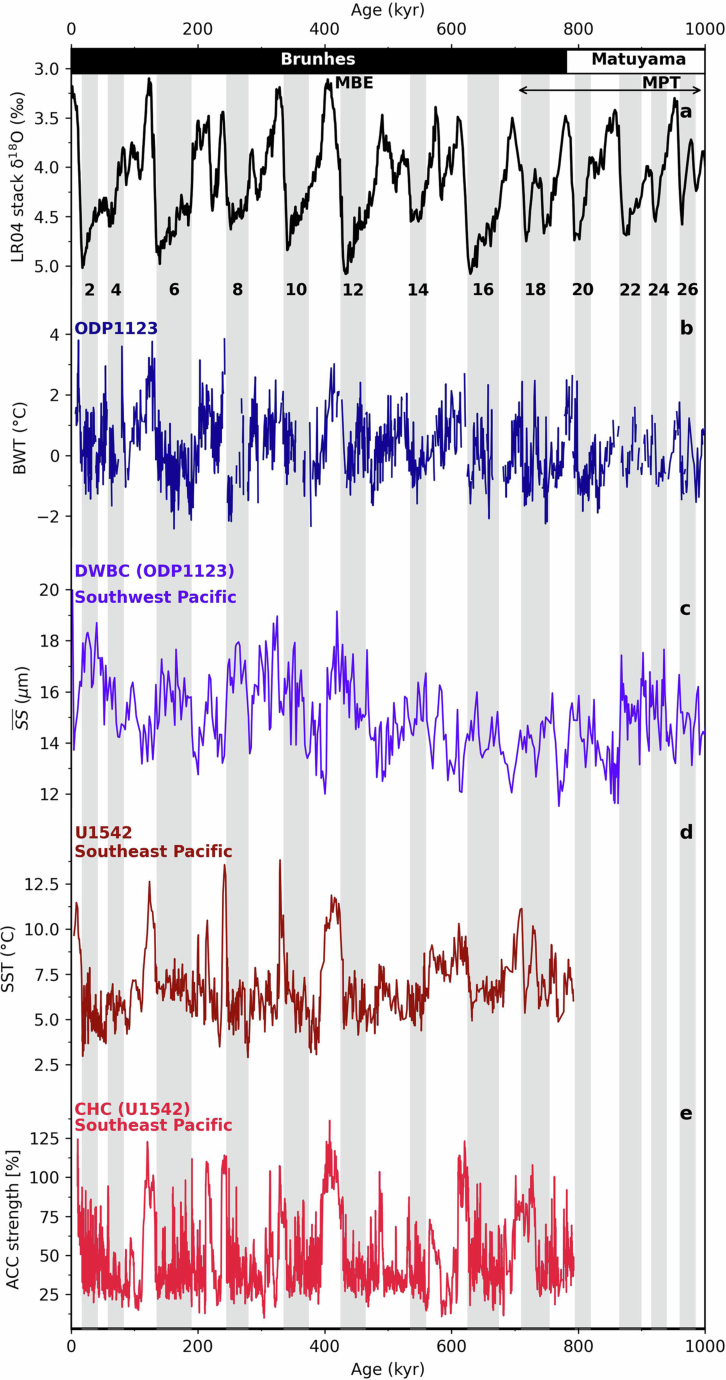
Evidence of northward transport of the ACC during glacial periods. **a**, Benthic foraminifera oxygen isotope stack^63^. **b**, Bottom water temperature (BWT) variations at site ODP1123 east of New Zealand^56^. **c**, Deep western boundary current (DWBC) variations at site ODP1123^55^. **d**. Sea surface temperature variations at site IODP U1542 in the Southeast Pacific^58^. **e**, Cape Horn Current (CHC) strength variations at site IODP U1542^58^.

Asymmetric changes in ACC flow also exerted fundamental influence on the global ocean circulation and interbasin exchanges. In the Indian sector, a stronger glacial ACC coincided with intensification of the deep western boundary current east of New Zealand, concurrent with a weaker ACC in the South Pacific^18,55^ (Fig. 2d,f,g). This configuration suggests enhanced northward export of glacial ACC into the tropical Pacific, consistent with pronounced cooling of deep water off New Zealand^56,57^ (Extended Data Fig. 10). Similarly, in the Drake Passage region, a weaker ACC during the ice ages is consistent with northward deflection of cold waters via the Humboldt Current into the South Pacific Gyre^16,17,30,58^. These patterns imply that reduced interbasin exchanges during glacial periods favoured CO_2_ sequestration by suppressing water masses mixing and upwelling^59,60^. Under warmer climatic conditions, an intensified ACC in the South Pacific promotes interbasin exchanges and thus facilitates the release of previously sequestered carbon to the atmosphere. Our reconstructions therefore provide robust evidence that future ACC intensification will probably increase interbasin connectivity and diminish the efficiency of the Southern Ocean as a sink for anthropogenic CO_2._

## Methods

### Sediment cores

Our study analyses six sediment records within the oceanic front system of the South Indian Ocean. These records are considered to reflect primarily the regional evolution of the ACC flow through time and for which local dynamics are deemed negligible. Four cores (MD11-3354, MD12-3394, MD12-3396 and MD84-551) were collected during RV *Marion Dufresne* MD185, 189 and MD38 cruises^65–67^. The remaining two piston cores (PS2609-1 and PS2606-6) were obtained from RV *Polarstern* cruise ANT-XI/4^68^.

Sites MD11-3354 (46° 13.87′ S, 87° 36.5′ E, 3,475 m water depth) and MD12-3396 (47° 43.88′ S, 86° 41.71′ E, 3,615 m water depth) are located in the central South Indian (Extended Data Fig. 1). These two sites sit at the east of the Kerguelen Plateau and the southern flank of the southeast Indian ridge. At present, MD11-3354 and MD12-3396 are located in the north of the SAF and lie in the dominant pathway of the ACC^69^. An ~40 m thick continuous sequence of Holocene to Middle Pleistocene (~980 kyr) sediments was obtained at Site MD11-3354. The sediment is characterized by carbonate-bearing to carbonate-rich diatom oozes, diatom-rich nannofossils and calcareous oozes.

Site MD12-3394 (48° 23’ S, 64° 35’ E, 2,320 m water depth) is located in the west of the Kerguelen Plateau (Extended Data Fig. 1). This site sits upstream in the ACC and west of the Kerguelen Plateau and in the Polar Front Zone between the SAF and the PF. Site MD84-551 (55° 0.5′ S, 73° 16.90′ E, 2,230 m water depth) is located in the west of the Fawn Trough and southwest of the Kerguelen Plateau (Extended Data Fig. 1). This site sits in the AZ between the PF and the SACCF.

Cores PS2609-1 (51° 29.9’ S, 41° 35.8’ E, 3,113 m water depth) and PS2606-6 (53° 13.9’ S, 40° 48.1’ E, 2,545 m water depth) are located in the west of Conrad Rise (Extended Data Fig. 1). These two cores lie in the AZ of the Southern Ocean, south of the PF, and are composed mainly of diatom ooze.

### Age models

For core MD11-3354, the oxygen isotopic composition of benthic foraminifera (*C. kullenbergi*) has been measured for the first 9 m at Laboratoire des Sciences du Climat et de l’Environnement (LSCE) on a GV Isoprime mass spectrometer (Extended Data Fig. 2). The δ^18^O measurements are reported versus Vienna Pee Dee Belemnite standard (VPDB) with NBS19 (National Bureau of Standards) standard at δ^18^O = –2.20‰, with a mean external reproducibility (1 s) of carbonate standards of ±0.06‰. Measured NBS-18 δ^18^O are –23.27 ± 0.10‰ VPDB. The reproducibility for *C. kullenbergi* δ^18^O (1 s) is ±0.11‰. The reflectance, L*, has been measured on board during the oceanographic cruise all along the core (Extended Data Fig. 2). For the past 190 kyr, the chronology has been established by correlating the benthic δ^18^O of MD11-3354 core to the δ^18^O LR04 stack^63^ using Analyseries software^70^. For the deeper part of the core, the stratigraphy has been made by correlating L* to the δ^18^O LR04 stack using Analyseries software^70^. The chronology is independently controlled by a sharp palaeomagnetic inclination transition from positive to negative values between 34.74 and 34.68 m, corresponding to the Brunhes–Matuyama boundary. These alignments converge on the LR04 δ^18^O stack with uncertainties of <3 ~ 5 kyr, ensuring that orbital-scale variability in sortable silt mean grain size remains unaffected.

We used the chronology of MD12-3394 from ref. ^60^, based on ^14^C radiocarbon dates on planktonic foraminifera and the correlation of reconstructed sea surface temperature by TEX86L to the Antarctic temperature stack. The age model of MD12-3396 we used was from ref. ^71^, based on ^14^C radiocarbon dates on planktonic foraminifera. The age model of core MD84-551 was taken from ref. ^29^, based on sea surface temperature and ^14^C radiocarbon dates. The age models of cores PS2609-1 and PS2606-6 were adapted from ref. ^72^, based on radiocarbon dates and biostratigraphic constraints. X-ray fluorescence records and biogenic opal and magnetic susceptibility signals were used to the refinement.

### Grain-size measurements

For sediment cores from the RV *Marion Dufresne* cruises, the detrital fraction of the sediments was isolated from the bulk sediment after removal of the carbonates by 10 ml hydrochloric acid (HCl, 10%) and the organic matter by 2 ml hydrogen peroxide (H_2_O_2_, 35%). The biogenic silica was removed with 40 ml sodium hydroxide (NaOH, 20%) under 85 °C for a period of 5–9 hours. A few drops of sodium hexametaphosphate (Na_6_[(PO_3_)_6_], 2%) was used to ensure complete desegregation of particles. For sediment cores from the RV *Polarstern* cruise, 1 g of freeze-dried sediment was wet sieved through a 63 μm net. After a one-week settling time, the samples were transferred to centrifuge tubes in a desegregating solution (Na_6_[(PO_3_)_6_]). The organic matter, carbonate and biogenic silica fractions were successively dissolved in a series of chemical treatments using 10% H_2_O_2_, 1 M acetic acid and 20% NaOH, respectively, separated by multiple steps of rinsing and vortexing. Remaining diatom frustules were physically removed by density separation using sodium polytungstate at a density of 2.25 g cm^−^^3^. Both procedures yielded very comparable results measured by laser diffraction analyser and SediGraph^17^. The grain-size measurements in this study were operated with a Malvern Panalytical’s mastersizer 2000/3000 laser diffraction particle-size analyser. The instrument precision of the Malvern 3000 for Silliker standard sample^73^ is less than 0.5% variation. Sortable silt mean grain size ($\overline{{\rm{SS}}}$) is defined as the mean grain size of the silt fraction (10–63 μm). Sampling resolutions are summarized in Supplementary Table 2.

### Spectral analyses

Spectral analyses were conducted by the Blackman–Tukey spectral power incorporated in the Analyseries software^70^. Linear trends were systematically removed and the values subsequently normalized. The frequency scale underwent a resampling process from 0 to 0.1, with an incremental step of 0.0002. We applied a Bartlett window with a bandwidth of 0.005 for these analyses (Extended Data Fig. 4). We then filtered the $\overline{{\rm{SS}}}$ record to extract its obliquity-paced signal (centred at 0.0244 ± 0.005 cycles per thousand years) so that its timing could be compared directly with the obliquity forcing (Extended Data Fig. 5).

### Modelling simulations

We use the simulations run with the NCAR CESM1.2^50,74^. This is a fully coupled global climate model, incorporating atmospheric, oceanic, land and sea-ice components. The NCAR CESM1.2 model offers a specific atmospheric resolution of 1° latitude by 1° longitude with 30 vertical levels and an oceanic resolution of 1° by 1° with 60 vertical levels. The NCAR CESM1.2 incorporates a spatiotemporally dynamic Gent–McWilliams eddy parameterization, providing a good first-order approximation of the effect of ocean eddy activity^75^. In this study, we use two obliquity simulations that set obliquity to the low (22.0798°) and high (24.4808°) extremes of the past 900 kyr. All other forcings are prescribed at preindustrial levels. Simulations were run for 500 years or longer. For more details, see ref. ^50^. We present regional average zonal winds across the combined Atlantic–Indian sector of the Southern Ocean (60° W to 160° E) and the South Pacific (160° E to 60° W; Fig. 4b and Extended Data Fig. 7).

## Online content

Any methods, additional references, Nature Portfolio reporting summaries, source data, extended data, supplementary information, acknowledgements, peer review information; details of author contributions and competing interests; and statements of data and code availability are available at 10.1038/s41561-025-01901-2.

## Supplementary information

Supplementary Tables Supplementary Tables 1 and 2.

## Data Availability

All relevant data in this paper are available via Zenodo (10.5281/zenodo.16945943)^76^. Simulation datasets for NCAR CESM are available at https://zenodo.org/records/1194490 (refs. ^50,77^). Geometric velocity datasets in Fig. 1 are available via NCEP Global Ocean Data Assimilation System (GODAS) at https://psl.noaa.gov/data/gridded/data.godas.html. Bathymetry background data in Extended Data Fig. 1 are available via the Global Multi-Resolution Topography (GMRT) synthesis at https://www.gmrt.org/index.php.

## Code availability

The analysis scripts generated for this study are highly customized to the specific data infrastructure and require specific explanation for use. The code is, however, available upon reasonable request from the corresponding author, who can provide the necessary support and documentation.
