# Supplementary material for: Zonally asymmetric changes in the Antarctic Circumpolar Current strength over the past million years
Source: Nat Geosci. 2026 Jan 29;19(2):201–8. doi: 10.1038/s41561-025-01901-2 (PMC12893906; doi:10.1038/s41561-025-01901-2)
Supplement: Supplementary file 1 — Supplementary Tables 1 and 2. [file 41561_2025_1901_MOESM1_ESM.pdf]

nature geoscience

Supplementary information https://doi.org/10.1038/s41561-025-01901-2

Zonally asymmetric changes in the Antarctic Circumpolar Current strength over the past million years

In the format provided by the authors and unedited

Supplementary Table. 1 | The compilation records of the ACC strength reconstruction

Ocean basin	Core	Lon [°E]	Lat [°N]	Depth [m]	Proxy	Strong flow speed	Reference
South Indian Ocean	MD11-3354	87.61	-46.23	3475	$\overline{SS}$	Glacial	This study
	MD12-3394	65.58	-48.38	2320	$\overline{SS}$	Glacial	This study
	MD84-551	73.28	-55.00	2230	$\overline{SS}$	Glacial	This study
	PS2609-1	41.60	-51.50	3113	$\overline{SS}$	Glacial	This study
	PS2606-6	40.80	-53.23	2545	$\overline{SS}$	Glacial	This study
	MD12-3396	86.69	-47.73	3615	$\overline{SS}$	Glacial	This study
	MD02-2589	25.26	-41.43	2660	$\overline{SS}$	Glacial	78
	MD02-2588	25.83	-41.33	2907	$\overline{SS}$	Glacial	21
	IODP U1475	25.26	-41.43	2669	$\overline{SS}$	Glacial	21
	SK 200/22a	45.07	-43.70	2730	Magnetic grain	Glacial	76
	MD00-2375G	86.74	-45.70	3485	Magnetic grain	Glacial	14
	MD11-3353	68.39	-50.57	1568	Magnetic grain	Glacial	26
	MD80-304	67.73	-51.67	1930	Benthic foraminifera	Glacial	79
	MD80-KK63	42.92	-51.93	2550	Benthic foraminifera	Glacial	79
	MD88-784	144.80	-54.20	2800	Benthic foraminifera	Glacial	79
	MD04-2720CC	71.37	-49.12	730	Benthic foraminifera	Glacial	79
	MD04-2721	71.13	-48.95	845	Benthic foraminifera	Glacial	79
SE Atlantic	ODP1090	8.90	-42.91	3702	$\overline{SS}$	Glacial	80
	MD07-3706	-14.23	-44.15	3770	$\overline{SS}$	Glacial	20
South Pacific	ODP1123	-171.50	-41.79	3290	$\overline{SS}$	Glacial	39
	PS97/085-3	-62.17	-58.35	3091	$\overline{SS}$ & ln(Zr/Rb)	Interglacial	17
	PS97/093-2	-70.27	-57.50	3781	$\overline{SS}$ & ln(Zr/Rb)	Interglacial	33
	MD07-3128	-75.57	-52.66	1032	$\overline{SS}$	Interglacial	16
	MR0806-PC09	-66.13	-55.71	684	$\overline{SS}$	Interglacial	16
	GC528	-58.06	-53.01	598	$\overline{SS}$	Interglacial	81
	IODP U1540	-114.84	-55.14	3580	$\overline{SS}$ & ln(Zr/Rb)	Interglacial	18
	IODP U1541	-125.43	-54.21	3604	$\overline{SS}$ & ln(Zr/Rb)	Interglacial	18
	PS75/076	-156.14	-55.53	3742	$\overline{SS}$ & ln(Zr/Rb)	Interglacial	18
	PS75/079	-157.24	-57.50	3770	$\overline{SS}$ & ln(Zr/Rb)	Interglacial	18
	PS75/083	-159.06	-60.27	3599	$\overline{SS}$ & ln(Zr/Rb)	Interglacial	18
	PS75/056	-114.79	-55.16	3581	$\overline{SS}$	Interglacial	80

SW Atlantic (Scotia Sea)	IODP U1537	-40.91	-59.11	3713	$\overline{SS}$	Interglacial	[19]
	IODP U1538	-43.36	-57.44	3131	$\overline{SS}$	Interglacial	[19]
	PS67/186-1	-41.32	-59.50	3671	$\overline{SS}$	Minor glacial-interglacial changes	[15]
	PS67/197-1	-44.10	-55.14	3837	$\overline{SS}$		
	PS67/205-2	-43.36	-56.70	3790	$\overline{SS}$		
	PS67/219-1	-42.47	-57.22	3619	$\overline{SS}$		
	PS67/224-1	-44.20	-57.94	2868	$\overline{SS}$		
	PS2319-1	-42.68	-59.79	4323	$\overline{SS}$		
	PS2514-1	-43.77	-53.67	2537	$\overline{SS}$		
	PC078	-45.02	-55.55	3840	$\overline{SS}$		
	TPC063	-48.04	-53.93	3956	$\overline{SS}$		
	TPC077	-45.47	-53.92	3774	$\overline{SS}$		
	TPC287	-36.65	-60.31	1998	$\overline{SS}$		
	TPC288	-37.96	-59.14	2864	$\overline{SS}$		

Supplementary Table. 2 Sampling intervals and sedimentation rates of sediment cores

Core	Sampling intervals (cm)	Sedimentation rates (cm/ka)	Average sed-rate (cm/ka)	Reference
MD11-3354	1~15	0.6~13.5	5.3	This study
MD12-3396	10~60	6.4~36.7	18.6	This study
MD12-3394	2~60	3.9~55.6	14.5	This study
MD84-551	1~29	1.8~28.6	6.5	This study
PS2609-1	11~300	7.4~46.3	18.6	This study
PS2606-6	16~200	4.6~32.7	11.1	This study
PS97/093	0.5~9	0.2~6.1	1.7	[33]
IODP U1540	1	0.7~12.4	2.8	[18]
IODP U1541	1	0.4~15.2	5.8	[18]
ODP1123	5~10	2.1~5.1	3.5	[39]
